# Inverse Kinematic Alignment in Robot-Assisted Total Knee Arthroplasty: A Simplified Surgical Technique

**DOI:** 10.1016/j.artd.2025.101798

**Published:** 2025-08-15

**Authors:** Ugonna N. Ihekweazu, Timothy B. Alton, Shawn O. Okpara, Philip G. Ghobrial, Corey F. Hryc

**Affiliations:** a Fondren Orthopedic Research Institute, Fondren Orthopedic Group, Houston, TX, USA; b Proliance Orthopedic Associates, Renton, WA, USA; c Department of Orthopedics, Baylor College of Medicine, Houston, TX, USA

**Keywords:** Inverse kinematic alignment, Robotic-assisted total knee arthroplasty, Gap balancing, Soft tissue balance, Joint line obliquity

## Abstract

Inverse kinematic alignment (iKA) is an emerging technique in total knee arthroplasty (TKA) that aims to restore the patients' native tibial joint line obliquity with femoral resections adjusted to balance the knee. By emphasizing joint line restoration and patient-specific balancing, iKA has gained interest as a potentially favorable alternative to traditional alignment techniques. This step-by-step surgical technique aims to outline the essential principles of iKA in robotic-assisted TKA. The method prioritizes an anatomic tibia resection and then a tensioner-based gap balancing technique to recreate natural kinematics. Since the technique is applicable to a wide range of patients and can be integrated to a variety of robotic platforms, iKA offers a promising pathway to standardize personalized alignment in TKA.

## Introduction

Total knee arthroplasty (TKA) effectively relieves pain and restores function in patients with advanced knee degeneration [1,2]. Despite rapid advancements in surgical techniques, applications, and implant designs, a significant number of TKA patients remain dissatisfied with their outcomes [3]. Efforts to identify and address reasons for dissatisfaction have focused on factors such as indications, soft tissue balance, and alignment [3]. Interest in alignment has grown due to its impact on knee kinematics and function [4,5]. Alternative alignment principles, including kinematic alignment, inverse kinematic alignment (iKA), and functional alignment, offer personalized implant positioning to potentially improve patient outcomes [6,7]. Among these, iKA has gained attention as a hybrid technique that can restore joint line obliquity and soft tissue tension while maintaining acceptable mechanical alignment limits.

Winnock de Grave et al. originally described iKA as a technique [8] that emphasizes an anatomic tibial resection to restore native tibial joint line obliquity. The anatomic coronal plane tibial resection is accomplished by aiming for equal medial and lateral tibial resections, compensating for cartilage and bone loss. At its core, iKA is tibia-first, gap-balancing method that aims to re-establish the knee's native kinematics within validated parameters, with a goal of enhancing clinical outcomes and patient satisfaction [8,9]. iKA distinguishes itself from other alignment strategies by accommodating a wider range of native coronal knee alignments, without requiring considerable modifications to the native coronal alignment angles [9]. This approach potentially offers benefits in the realm of soft tissue balance and functional outcomes, as it aims to closely replicate both the patient's natural knee anatomy and kinematics [9,10].

A notable concern regarding the widespread adoption of alternative alignment strategies, such as iKA, is the potential challenge of consistently achieving precise targets using manual instruments. As a result, there has been considerable growth in the implementation of enabling technologies in TKA. Between 2015 and 2020, the utilization of robotic-assisted TKA (RA-TKA) increased by 601.2% and has been associated with a reduction in the duration of hospitalization, postoperative complications, and postoperative opioid use [11]. In terms of precision, RA-TKA has been shown to be superior to manual techniques in achieving accurate coronal and sagittal alignment with fewer outliers [[12], [13], [14], [15], [16]].

The utilization of RA-TKA is anticipated to increase as current trainees become more proficient with these techniques. With the expanding prevalence of RA-TKA and the growing interest in contemporary alignment strategies, there is a need for standardized instruction to expedite the learning curve and optimize surgeon proficiency. This step-by-step surgical technique aims to outline the essential principles of iKA in RA-TKA and provide a framework for trainees and surgeons to incorporate iKA RA-TKA into their surgical practice.

## Surgical technique

**Figure 1:**
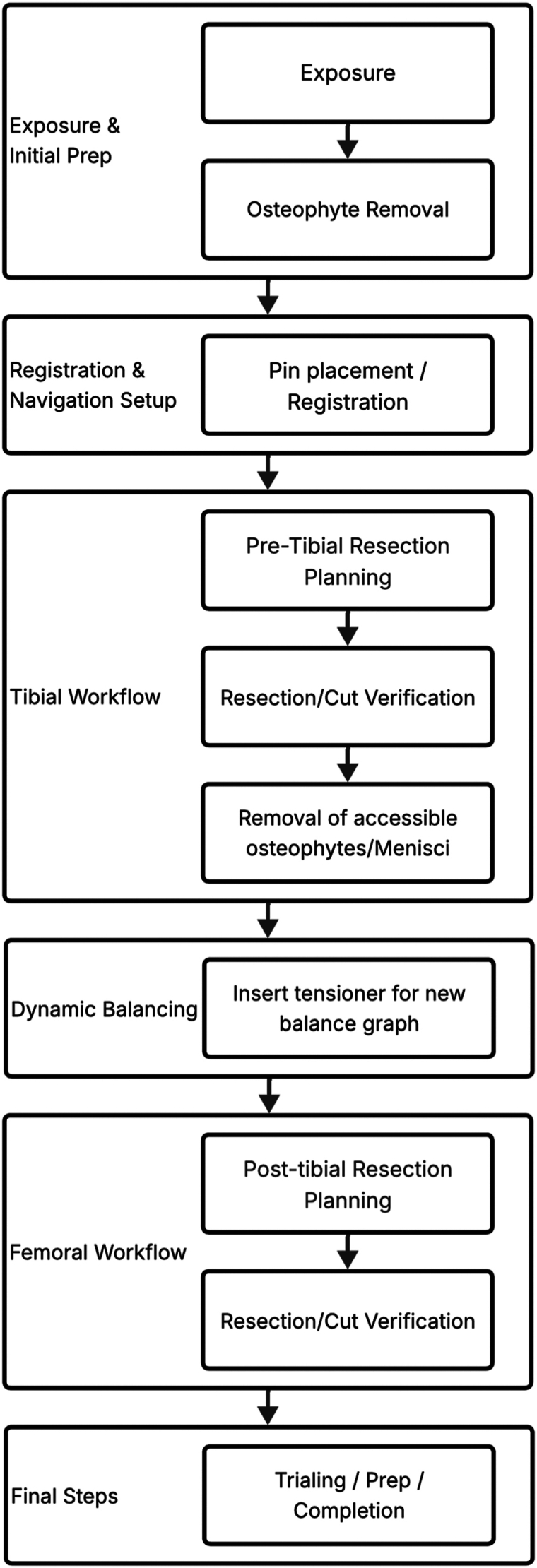
Surgical workflow for RA-TKA.

The procedure follows a standard RA-TKA technique (Fig. 1). Briefly, registration is done using intraoperative landmarks. These landmarks are validated before assessing the patient's native soft tissue tension. The tibial resection is planned to match the coronal plane anatomy. After the tibial cut, the meniscus and accessible osteophytes are removed, and a tensioning device is used to re-establish soft tissue balance. Finally, femoral component adjustments ensure the knee remains balanced throughout its range of motion with a focus on minimizing soft tissue releases.

A key advantage of this technique is its simplicity and versatility, allowing it to be applied to various phenotypes and deformities. Fundamentally, the technique aims to restore the patient's native joint line obliquity while ensuring that the knee remains balanced throughout a full range of motion. An additional benefit of this technique is surgical workflow efficiency where recuts on the tibial or femoral side are quite rare. The following sections will provide detailed instructions and the rationale behind the restorative tibial resection, the use of the tensioning device, and the final step of balancing the knee with the femoral component.

### Tibial planning and resection

To perform an anatomical tibial resection in the coronal plane, careful registration and planning are essential. Achieving an anatomic coronal plane resection involves ensuring equal medial and lateral tibial resections while accounting for cartilage and bone loss. This step can be executed using 1 of 2 techniques: measured resection or preoperative planning. With image-based platforms that reference bone, the measured resection technique requires the surgeon to aim for equal medial and lateral bony resections of the tibial plateau [8]. The thickness of the resection is measured from the middle of the medial and the middle of the lateral plateau, which coincides with the contact point of the femoral condyle.

**Figure 2:**
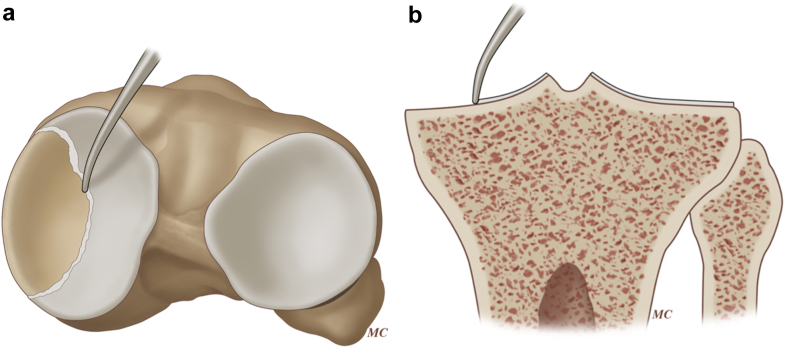
The tidemark, defined as the transition zone between articular cartilage and subchondral bone, serves as the proximal tibial reference point during resection. In both the axial (a) and coronal (b) views, the probe is shown positioned at the transition point (Illustration by Michael Cooley).

**Figure 3:**
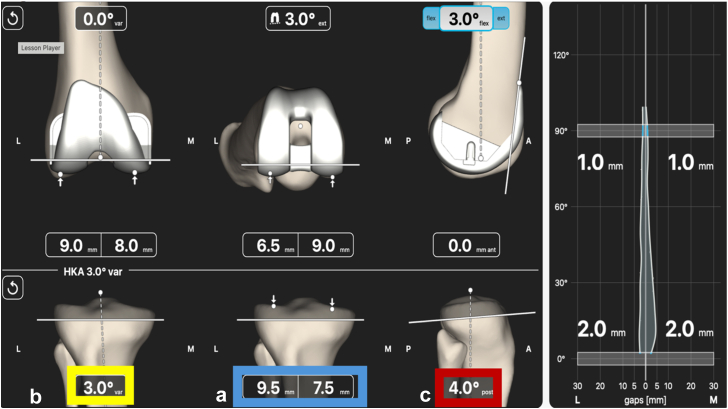
The pretibial resection planning. (a) Measured resection technique, indicated by the blue border, where the tidemark was used during the registration process and a 2 mm difference between the medial resection and the lateral resection is planned to compensate for cartilage wear. (b) Preoperative MPTA measurement, indicated by the yellow border, where 3° was measured on preoperative imaging and directly put into the software during the planning phase. (c) Anatomic slope matching, indicated by the red border, the patient’s medial tibial slope in this sample case was determined to be 4°. Once the coronal and sagittal parameters for the tibial resection are confirmed, the surgeon may then adjust the tibial resection depth to accommodate for the planned polyethylene component. Once confirmed the tibial resection is completed.

Alternatively, surgeons using platforms that reference cartilage may use the tidemark registration approach. This is an intraoperative technique where the surgeon uses the tidemark for the point of reference during the registration process (Fig. 2 – illustration). When the tidemark is used as a referenced point, an assumption of 2 mm of cartilage loss can be made [17,18]. If the opposing compartment has minimal cartilage wear, then coronal plane tibial resection planning is based on a 2 mm difference between the compartments (Fig. 3a). For a conventional varus knee, if the planned resection is 9 mm for the unworn lateral compartment, the medial compartment resection should be 7 mm when referencing the tidemark. This approach ensures equal medial and lateral tibial resection. While the concept of a uniform 2-mm cartilage thickness is cornerstone of kinematic alignment techniques, a recent study suggests that unworn cartilage thickness may be highly variable [19].

**Figure 4:**
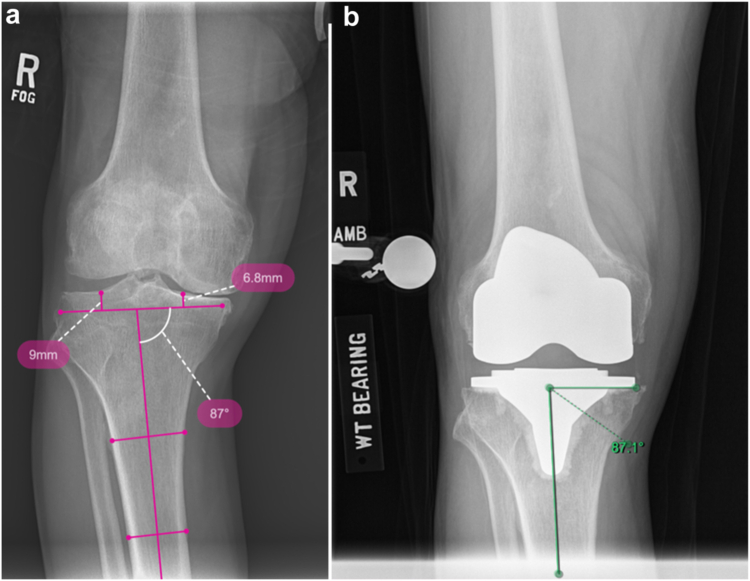
An alternative method involves preoperative templating of the MPTA and direct execution of this measured angle intraoperatively. (a) In this case example, the preoperative MPTA was determined to be 3° of varus. (b) Postoperative radiographs confirm restoration of the 3° tibial joint line resection angle.

In cases of significant bone loss, the tidemark may shift laterally along the lateral aspect of the medial tibial plateau or toward the tibial spine. This can misrepresent the native dwell point of the medial tibial plateau and result in an under-resection medially. Therefore, an alternative method involves preoperative templating of the medial proximal tibial angle (MPTA) (Fig. 4). The MPTA is defined as the angle formed by the anatomic axis of the tibia and the orientation of the proximal tibial joint line, which is a line connecting the medial and lateral tibial plateaus. This angle can be measured using preoperative imaging, ideally full-length hip-knee-ankle radiographs, and directly planned during surgery when preparing for the tibial resection (Fig. 3b). Regarding tibial resection boundaries, the aim is to restore the MPTA to a clinically acceptable range of 84° (varus) to 92° (valgus), which corresponds to native knee alignment in 93% of cases [20].

It is important to note that if full-length radiographs are unavailable and knee radiographs are used for preoperative measurement of the MPTA, as shown in Figure 4, this may introduce error into the procedure because the tibial mechanical axis referenced by robotic platforms does not always align with the tibial anatomic axis. Finally, the MPTA technique is not suitable for use in scenarios where the anatomic axis and the mechanical axis of the tibia clearly differ, such as in cases involving extra-articular tibial deformities.

Regarding the planning of the tibial resection in the sagittal plane, 2 methods are available. The first is a conventional technique, where the slope is determined based on the presence or absence of the posterior cruciate ligament (PCL). The second method involves matching the native slope by following the natural slope of the medial tibia plateau. In conventional slope planning, a typical slope of 3° is planned when the PCL is removed [21], whereas a slope of approximately 5° is planned with the retention of the PCL [22]. Advocates for this technique contend that implant design should be considered in postoperative kinematics, suggesting that adherence to manufacturer recommendations regarding slope may result in more favorable kinematic outcomes [21,22]. Alternatively, anatomic matching of the slope can be achieved using RA-TKA software to replicate the patient's native medial proximal tibial slope angle. Slope matching may be performed on a computed tomography–based platform by aligning the tibial component with the patient's native sagittal anatomy [23]. Alternatively, the patient's slope can be observed intraoperatively by utilizing a navigated probe, which can be visualized in real time on the planning screen to facilitate alignment of the tibial component parallel to the native reference (Fig. 3c). Some studies indicate that recreating the anatomic tibial slope may improve postoperative flexion and overall knee function [24,25].

Once the coronal and sagittal plane resection angles are established, the resection depth is determined based on the target polyethylene liner thickness. Axial planning of the tibial resection should follow the patient's native anterior–posterior axis, also referred to as Akagi's line [26], which aligns with the trajectory of the PCL, anterior cruciate ligament, and the medial third of the tibial tubercle. Deviations from the anterior–posterior axis can lead to uneven distribution of slope either medially or laterally during tibial resection, potentially affecting coronal plane alignment and flexion gap stability [27].

**Figure 5:**
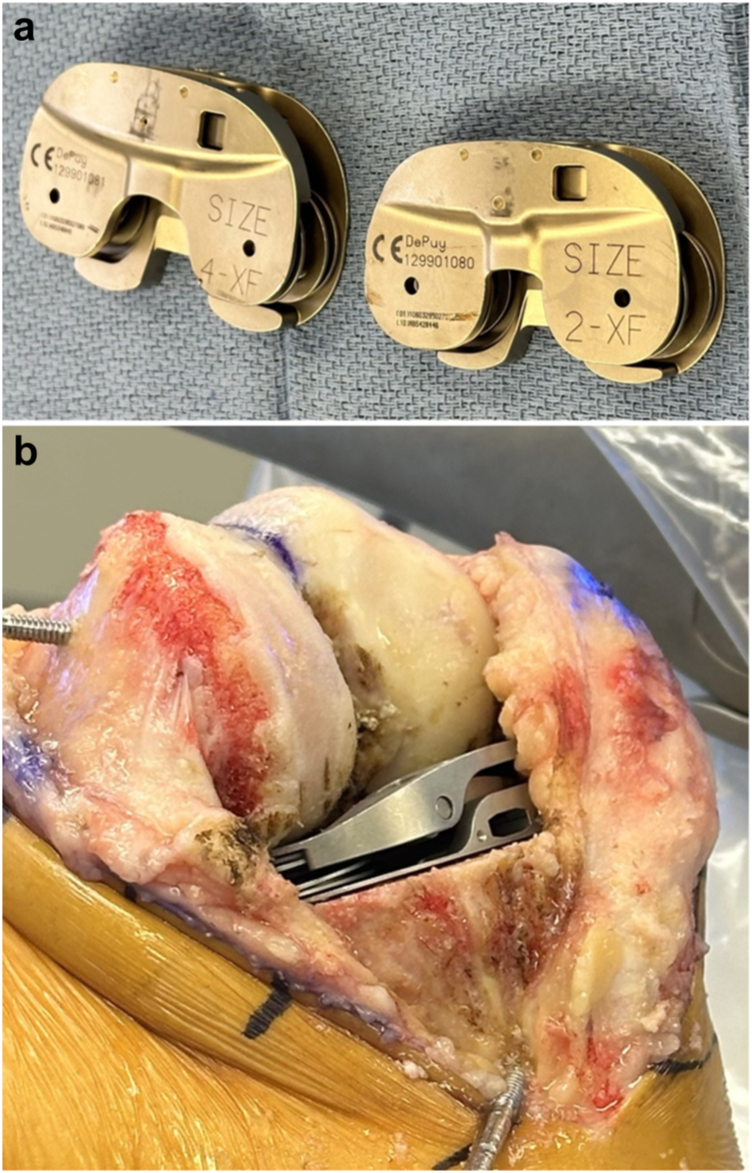
Spring-loaded tension device. (a) Device shown in 2 sizes. (b) Device inserted into flexed knee following tibial cut.

Once the tibial cut is planned, the tibial resection is completed, and the cut bone is removed along with all remaining accessible osteophytes and the menisci. The surgeon should verify the accuracy of the cuts using the software once each cut is made. Next, a spring-loaded tensioning device (Fig. 5), which delivers between 86 to 113 newtons per compartment, is inserted into the flexed knee. Alternatives to this spring-loaded device include a digital tensioner [28] or an appropriately sized spacer block or trial liner. If a spacer block or trial liner is utilized, the surgeon should ensure that uniform load is distributed to the soft tissues throughout a range of motion. The knee is then moved through its range of motion with the tensioning device in place, and a new soft tissue balance graph is created in extension and at 90°.

### Femoral planning and resection

**Figure 6:**
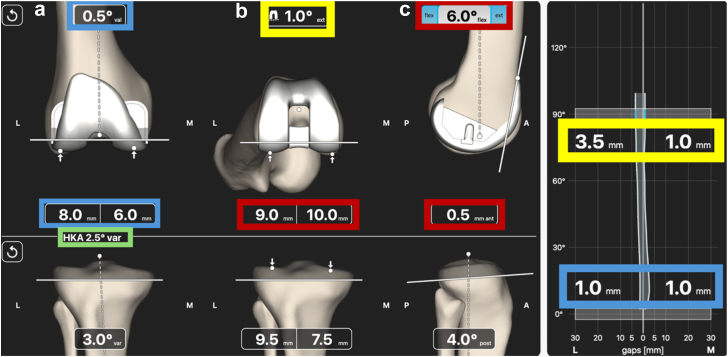
Post-tibial resection planning. (a) Femoral coronal plane planning (Blue borders). Zero point five degrees of valgus was applied to achieve equal medial and lateral extension gaps. Note the 2-mm difference between the medial and lateral resection, indicating there was cartilage loss encountered on the medial distal femoral resection for this sample case. The final hip-knee-ankle angle is confirmed with this step (Green border). (b) Femoral Axial plane planning (Yellow border). The femoral component was internally rotated by 2° from a standard 3° starting point, resulting in a planned posterior condylar resection ie, now 1° external to the posterior condylar axis. The result is an asymmetric flexion space, with the medial gap tighter than the lateral gap by 2.5 mm. (c) Femoral component planning is completed with sagittal plane adjustments (Red Border). With anterior referencing, femoral component flexion closes the flexion gap and reduces the posterior condylar resection. Once appropriate balance has been achieved, the femoral cuts are completed.

Once the tibial resection is performed and the final soft tissue envelope has been established, the surgeon completes the final planning step by balancing the knee with multiplanar adjustments of the femoral component. For the femoral coronal plane, adjustments only affect the extension gap as alignment and cut depth can be modified. Introducing varus in the coronal plane will widen the medial extension gap, whereas increasing valgus will widen the lateral extension gap. The objective for the extension gap is to achieve measurements that are relatively similar, typically within 1 mm of each other (Fig. 6a). Additionally, the resection thickness of the distal femoral cut should correspond to the thickness of the femoral component. Once femoral coronal plane adjustments have been finalized, both the hip-knee-ankle angle and the extension gaps are confirmed (Fig. 6a).

Next, adjustments to the femur are performed in the axial plane; alterations in the axial plane affect the flexion gap alone. The goal for the axial plane is to match the native anatomy and knee kinematics which follows a screw home mechanism, whereby, the lateral femoral condyle rolls back as the knee flexes [29]. To facilitate this, the medial flexion gap should be tighter than the lateral flexion gap, often requiring internal rotation of the femoral component relative to what is customary in mechanically aligned TKA’s that utilize a measured resection technique, where the femoral component is aligned 3° externally rotated relative to the posterior condylar axis. Since the aim is to closely restore anatomy, the thickness of the medial and lateral posterior condylar bone cuts should match implant thickness. Most wear patterns show minimal posterior condylar cartilage loss [30], so the aim for this step is to achieve equal medial and lateral posterior condylar resection. Matching the posterior condylar anatomy typically results in asymmetric flexion gaps, where the medial gap is narrower than the lateral gap. It is common to internally rotate the femoral component to achieve soft tissue balance with iKA. The ability and desire to adjust femoral rotation to achieve soft tissue balance is fundamental to this technique. Many have shown that alternative femoral rotation when performed in conjunction with iKA techniques does not have a negative impact on patellafemoral tracking (Clatworthy 2020). Some surgeons may institute boundaries around maximum allowed femoral rotation, but these are based on historical principles and are being challenged with modern techniques and more data around iKA [31].

Regarding the femoral sagittal plane, when the pivot point is at the anterior cortex, alterations affect primarily the flexion gap (Fig. 6c). Component flexion and extension, as well as anterior and posterior positioning, can be modified. Flexion and extension of the femoral component result in a symmetric decrease and increase of flexion gap space, respectively. Similarly, anterior and posterior positioning cause a symmetric increase and decrease of flexion gap space, respectively. Femoral upsizing and downsizing can also be leveraged to address balancing the flexion space. Sagittal component adjustment of the femoral component should be used to finalize the relative thickness of the posterior condylar bone cuts and to ensure there are no anterior compartment issues, such as notching or overstuffing. Once planning is confirmed, the femoral cuts are conducted in the usual manner. Care is taken to verify the accuracy of the cuts with the software once each cut is completed. Following the resections, the procedure is finished following the standard steps of trialing, prepping, and implantation of the final components. If it is determined that the knee is not sufficiently balanced during the trialing phase, adjustments can be made by performing additional soft tissue releases or by recutting the tibia to achieve the desired balance.

This workflow is simple with consistent steps for all deformities and phenotypes. The steps are an anatomic tibial resurfacing followed by soft tissue tensioning and achieving soft tissue balance though the femoral bone resections minimizing soft tissue releases. Femoral or tibial recuts are rare making this an efficient and reproducible technique.

## Discussion

Herein we describe an RA technique for iKA in TKA using a spring-loaded tensioning device. The method involves simple, reproducible steps, suitable for all TKA cases. It aims to restore the joint line obliquity through an anatomic tibial resection and comprehensive soft tissue balancing based on gap data from the robotic platform.

As interest in alternative alignment platforms continues to grow among arthroplasty surgeons, individual philosophies such as functional alignment, kinematic alignment, and iKA have emerged. iKA distinguishes itself from other techniques by aiming to restore the tibial anatomy and native joint line obliquity while also precisely balancing the soft tissues throughout a range of motion.

Several studies have highlighted the potential benefits of iKA. In a retrospective review, Winnock de Grave et al. reported that iKA resulted in higher patient satisfaction and improved Oxford Knee Scores compared to adjusted mechanical alignment at the 12-month follow-up [32,33]. A few studies suggest that iKA may be more effective in restoring the natural alignment of the knee. Keyes et al. reviewed preoperative and postoperative alignment in 79 patients undergoing TKA and found that the iKA technique reliably restored the constitutional alignment in a majority of the cases [34]. Another by Winnock de Grave et al demonstrated that iKA better accommodated native coronal knee alignment compared to anatomical mechanical alignment and restricted kinematic alignment [9]. Similarly, Murgier et al. evaluated 287 TKAs performed using a patient-specific alignment and balanced technique and found that variable femoral component rotation had no significant effect on clinical outcomes, including Oxford Knee Scores, WOMAC, KOOS JR, Forgotten Knee Score, or patient satisfaction [31].

Biomechanically, iKA has been shown to restore the original tibial joint line obliquity without increasing knee adduction moments, which is crucial for maintaining normal knee function and minimizing the risk of implant failure [9]. Finally, Genestoux et al. examined the effect of iKA on patellofemoral kinematics and reported that specific patellar clinical scores were excellent at 1 year [35].

### Limitations

This technique for TKA is not without limitations. Firstly, soft tissue balance graphs are based on gap data, which may not accurately present opportunities for optimal soft tissue balance. iKA techniques are dependent on advanced navigation systems that provide this intra operative gap data. Some contend that intraoperative pressure sensors are able to provide more objective feedback leading to more balanced knees and enhanced kinematics [36,37]. Secondly, the tensioner utilized in this technique is spring-loaded. Although the Newton force applied to each compartment is intended to be uniform throughout a range of motion, ensuring consistency across all knee phenotypes, deformities, and patient populations is challenging for the surgeon. Also, the optimal force to be applied for a specific patient, if variable force options are available, is debatable.

Furthermore, the goals for soft tissue balance assume that patients generally prefer relatively equal extension gaps and asymmetric flexion gaps, where the medial flexion gap is tighter than the lateral gap. While this distribution of soft tissue tension is intended to replicate native knee kinematics, there is no evidence, to our knowledge, supporting the uniform application of these gap preferences across the entire patient population.

Ultimately, further research is required to better understand the appropriateness of this technique for specific patient populations and determine if other methods for achieving soft tissue balance might be more effective.

## Summary

RA iKA is a straightforward and consistent method applicable to various phenotypes and deformities encountered in primary TKA. This technique aims to restore the native joint line obliquity while achieving a well-balanced TKA construct. More research is needed to clearly define soft tissue goals for specific patients.

## Conflicts of interest

Ugonna N. Ihekweazu is on the speakers' bureau, is a paid consultant for, and receives research support from J&J Med Tech; owns stock in Revel AI, Visie, Romtech; receives fellowship support from AAHKS; and is on the AAHKS board of directors. Timothy B. Alton is on the speakers' bureau of and is a paid consultant for J&J Med Tech and Solventum. Corey F. Hryc receives research support from J&J Med Tech. The other authors declare no potential conflicts of interest.

For full disclosure statements refer to https://doi.org/10.1016/j.artd.2025.101798.

## CRediT authorship contribution statement

**Ugonna N. Ihekweazu:** Writing – review & editing, Writing – original draft, Visualization, Project administration, Methodology, Investigation, Conceptualization. **Timothy B. Alton:** Writing – review & editing, Validation, Methodology, Investigation. **Shawn O. Okpara:** Writing – review & editing, Writing – original draft, Validation, Investigation. **Philip G. Ghobrial:** Writing – review & editing, Writing – original draft, Investigation. **Corey F. Hryc:** Writing – review & editing, Writing – original draft, Visualization, Validation, Supervision, Project administration.

## Appendix A Supplementary data

Conflict of Interest Statement for Alton

Conflict of Interest Statement for Ghobrial

Conflict of Interest Statement for Hryc

Conflict of Interest Statement for Ihekweazu

Conflict of Interest Statement for Okpara
