# Supplementary material for: Inverse Kinematic Alignment in Robot-Assisted Total Knee Arthroplasty: A Simplified Surgical Technique
Source: Arthroplast Today. 2025 Aug 15;35:101798. doi: 10.1016/j.artd.2025.101798 (PMC12391249; doi:10.1016/j.artd.2025.101798)
Supplement: Conflict of Interest Statement for Ihekweazu [file mmc4.pdf]

CONFLICT OF INTEREST STATEMENT

American Association of Hip and Knee Surgeons

(Adopted from the American Academy of Orthopaedic Surgeons disclosure statement)

The following form **must be filled out completely and submitted by each author (example, 6 authors, 6 forms). All items require a response. If there is no relevant disclosure for a given item, enter "*None*."**

Manuscript Title : **Inverse Kinematic Alignment in Robot-Assisted Total Knee Arthroplasty: A Simplified Surgical Technique**

1. Royalties from a company or supplier (The following conflicts were disclosed)

2. Speakers bureau/paid presentations for a company or supplier (The following conflicts were disclosed)

   J&J Med Tech

3A. Paid employee for a company or supplier (The following conflicts were disclosed)

   None

3B. Paid consultant for a company or supplier (The following conflicts were disclosed)

   J&J Med Tech

3C. Unpaid consultants for a company or supplier (The following conflicts were disclosed)

   None

4. Stock or stock options in a company or supplier (The following conflicts were disclosed)

   Revel AI, Visie, RomTech

5. Research support from a company or supplier as a Principal Investigator (The following conflicts were disclosed)

   J&J Med Tech

6. Other financial or material support from a company or supplier (The following conflicts were disclosed)

   Fellowship Support: AAHKS

7. Royalties, financial or material support from publishers (The following conflicts were disclosed)

   None

8. Medical/Orthopaedic publications editorial/governing board (The following conflicts were disclosed)

   None

9. Board member/committee appointments for a society (The following conflicts were disclosed)

   AAHKS Board of Directors

Each author must sign AND print or type his/her name, date and submit a separate form

In addition, one BLINDED Conflict of Interest form (no author names used) should be submitted per manuscript with all author disclosures.

UGONNA IHEKWEAZU [signature] 4/25/25

Author Name (Print or Type) Author Signature Date
